# Optimization of e-commerce logistics service quality considering multiple consumption psychologies

**DOI:** 10.3389/fpsyg.2022.956418

**Published:** 2022-09-05

**Authors:** Meng Ma, Lu Shen, XuanQing Sun

**Affiliations:** College of Finance and Economics, Xinxiang Vocational and Technical College, Xinxiang, China

**Keywords:** multiple consumer psychology, e-commerce, logistics services, optimization research, distribution personnel

## Abstract

This work is developed to improve the current quality of e-commerce logistics services. From the perspective of multiple consumer psychology, based on e-commerce, consumer psychology, and other related theories, vegetable e-commerce B is selected as the research object. The commodity quality, accuracy, and timeliness of commodity distribution and other factors of e-commerce B are discussed through questionnaire survey. Then, according to customers’ opinions about “e-commerce B’s distribution and professional aspects that need to be improved,” the research is conducted. Finally, the direction of follow-up optimization is proposed from four different perspectives of multiple consumption psychology. The research results show that more than 89% of the surveyed customers believe that e-commerce B does a good job in terms of commodity quality, accuracy, and timeliness of commodity distribution, and has a high level of logistics service. However, 26.34% of customers hope that e-commerce B can strengthen the protection of personal privacy, 24.97% hope that the platform can add “delay insurance” for goods, and 39.54% hope that the logistics information of purchased goods can be updated in real time. Therefore, e-commerce B needs to be optimized and improved continuously in the future development. Therefore, research on the optimization of logistics service quality of e-commerce is performed under multiple consumption psychology, which provides certain help for the rapid development of subsequent e-commerce.

## Introduction

With the arrival of the 5G era and the rise of e-commerce logistics, more and more people are choosing to shop on their phones or computers. Compared with traditional shopping methods, online shopping has advantages of convenience and low transaction cost (Jiang et al., 2021). However, in the actual development process, there are still many problems, such as the storage of fresh goods, distribution, and quality of goods. These problems will undoubtedly reduce customer experience. Therefore, how to ensure the interests of enterprises while not affecting customers’ experience is one of the problems that all e-commerce platforms are generally solving (Qin et al., 2021; Vasić et al., 2021).

After studying different types of e-commerce platforms, Zhang et al. (2021b) found that fresh e-commerce has the greatest development potential. Because fresh has the characteristics of high frequency, rigid demand, and full population coverage, it is a high-quality flow entrance. However, due to the lack of relevant e-commerce development experience and many difficulties in the distribution of fresh goods, there are not many enterprises that can make profits from this business at present (Zhang et al., 2021b). Lin et al. (2022) believed that among the three major logistics service modes, outsourcing logistics has three major problems, namely hidden security risks, low service quality, and weak supervision. To improve the development status of outsourcing logistics, some strategies are proposed, such as identifying customer perception service indicators, clarifying the responsibilities of each subject, mutual penetration of multiple outsourcing logistics services, and collaborative development with new e-commerce retail (Lin et al., 2022). Ding and Zhao (2021) studied the efficiency of logistics distribution, taking more than 100 orders of a supermarket chain in Taiyuan as the research object, transforming and clustering them with K-means algorithm, and then optimizing the path. It aims at shortest route, highest reward, highest customer satisfaction, and lowest punishment cost. Finally, the feasibility of the above methods in solving supermarket order allocation and path optimization is verified (Ding and Zhao, 2021). Su (2021) believed that the service level established by e-commerce enterprises should be different from the offline service level. In the process of e-commerce shopping, there is little direct interaction between people, so factors such as politeness, cleanliness, friendliness, care, commitment, and flexibility are not critical in e-commerce services. However, accessibility, communication, credibility, understanding, appearance, availability, and other determinants are applicable to both offline and e-commerce (Su, 2021). Wang C. N. et al. (2021) believed that the quality of e-commerce logistics service depends on the actual perception of customers, rather than the service perceived by enterprises themselves. Regular satisfaction survey is what every e-commerce enterprise should adhere to (Wang C. N. et al., 2021). Wang Y. et al. (2021) established 20 indicators to evaluate the service quality based on the development characteristics of E-commerce logistics services in China. The results showed that the overall service level is average, but there are still many problems in details, such as after-sales management and delivery personnel selection (Wang Y. et al., 2021). From different perspectives, the literatures of the above scholars studied the service quality, hidden dangers, and logistics distribution efficiency of e-commerce. They influenced and learned from each other, aiming to promote the healthy and long-term development of e-commerce.

To sum up, from the perspective of multiple consumption psychology, questionnaire survey is used to study the commodity quality and delivery timeliness of E-commerce enterprise B. Then, it states the areas that customers think need to be improved in delivery professionalism. Based on the above results, it gives corresponding opinions from four different perspectives of multiple consumption psychology. The innovation of this work lies in the evaluation of the service quality of e-commerce logistics through questionnaire survey, and then feasible opinions on the existing problems are put forward. The overall logic is clear. At the same time, it also enriches the academic theoretical research in this field and provides methodological reference for future research on e-commerce logistics service optimization.

## Relative concepts and methods

### Multiple consumption psychology

**FIGURE 1:**
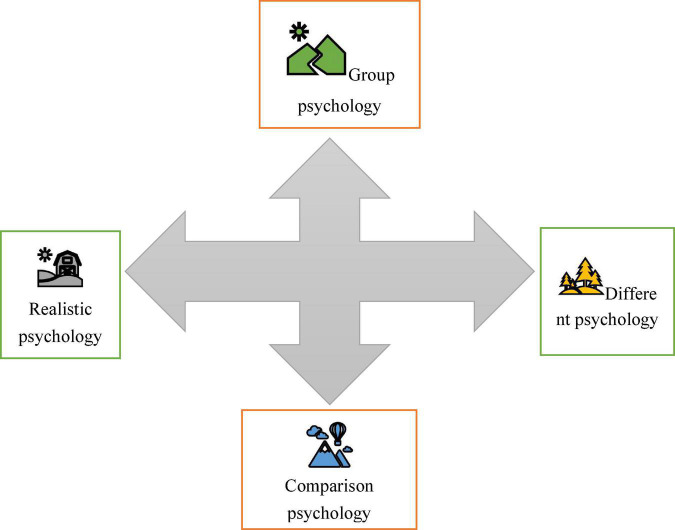
Consumer psychology.

Consumer psychology refers to the psychological characteristics and process of psychological activities shown by consumers in their consumption activities. It is all the psychological activities of consumers and the consumption behaviors generated therefrom, including that the consumers observe commodities, collect commodity information, choose commodity brands, make purchase decisions, use commodities to form psychological feelings and experience, and provide information feedback to production and operation units (Guan, 2021). Consumer psychology is roughly classified into four types (Figure 1).

Consumer psychology is roughly classified into four types, namely, conformity psychology, seeking difference psychology, comparison psychology, and realistic psychology. Conformity psychology is imitative and blind. Whether consumers should follow the crowd should be analyzed in a case-by-case manner. In short, blind conformity is not desirable. The characteristic of seeking difference is the pursuit of originality and distinctiveness. Its advantage is that it can promote the emergence of new technologies and new products, while its disadvantage is that the presentation of individuality should not only consider social recognition but also consider the cost. It is not worth advocating excessive novelty to show uniqueness (Bihua, 2021). The characteristic of comparison psychology is face consumption, which is still not desirable. Realistic consumers tend to consider many factors when choosing goods. Such consumer psychology can choose commodities according to their own needs, which is a kind of rational consumption (Wang X. et al., 2021).

**FIGURE 2:**
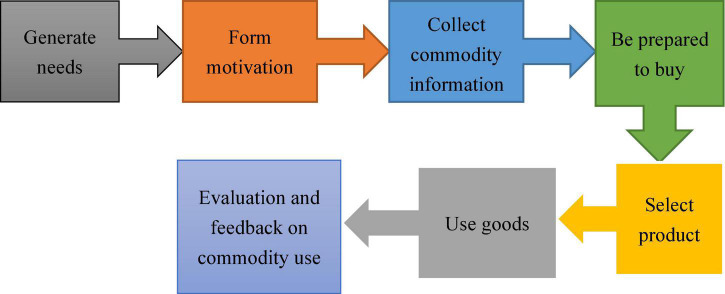
Consumer psychological process.

The psychological process of consumers is mainly divided into seven stages, as illustrated in Figure 2.

**FIGURE 3:**
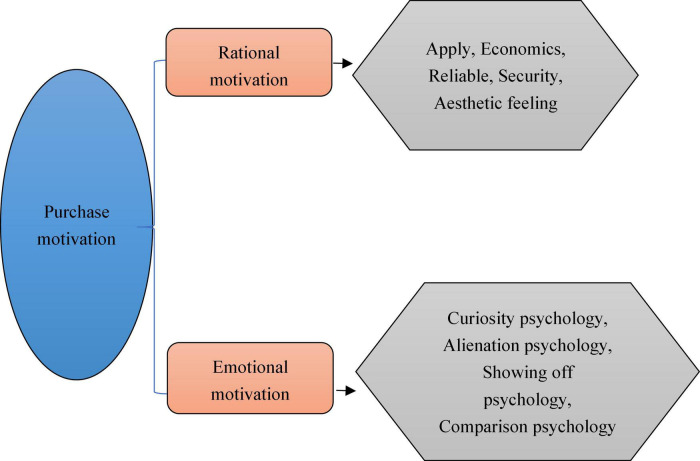
Consumers’ purchasing motivation.

Consumers’ purchase of goods is mainly influenced by purchase motivation, which is to guide customers’ purchase activities to a certain target to meet their purchase intention and impulse (Elder and Krishna, 2022). Such purchase intention and impulse are very complex and unpredictable psychological activities. From the perspective of its performance, consumers’ purchase motivation is classified into two categories (Figure 3).

From Figure 3, consumers’ purchase motivations are rational motivation and emotional motivation. Rational motives include applicability, economy, reliability, safety, esthetic feeling, convenience of use, convenience of purchase, and after-sales service (Grier et al., 2022). Emotional motivation is not just irrational motivation; it is mainly the purchase intention and impulse produced by social and psychological factors. It is difficult to establish an objective standard for emotional motivation, which is mainly caused by curiosity, alienation, showing off, comparison, conformity, and respect (Ozanne et al., 2022).

### The electronic commerce

Electronic commerce is a business activity centered on commodity exchange by means of information network technology. Among them, “electronic” is a kind of technology and a means, while “commerce” is the core purpose, and all means are generated to achieve the purpose (Saad, 2021). The e-commerce division uses computer technology, network technology and other modern information technology to carry out relevant work. The components of e-commerce are mall, consumer, product, and logistics (Donthu et al., 2021; Li, 2022).

The traditional e-commerce logistics mode is mainly composed of three types, namely, third parties, self-management, and outsourcing. Third-party logistics is a professional logistics company independent of buyers and sellers that undertakes entrusted logistics services in the form of long-term cooperation (Kim et al., 2021). It is necessary to solve the logistics problems for enterprises in an all-around way so that enterprises’ products quickly move to the market, thus reducing logistics costs and improving economic benefits. Third-party logistics does not participate in the buying and selling of goods but provides personalized logistics agency services for customers (Weber et al., 2021). It is also the main logistics mode of e-commerce, which is more convenient, fast, and high-speed (Forehand et al., 2021). The self-management mode is the process in which an enterprise sets up its own logistics distribution system and manages its logistics distribution. At present, there are two main types of self-management enterprises in China: e-commerce companies with abundant financial strength and a large business scale and large-scale manufacturing enterprises (Patrick and Hollenbeck, 2021; Schwarz et al., 2021). The logistics cooperation alliance is established by outsourcing logistics enterprises based on mutual agreements, such as manufacturing and sales enterprises. Enterprises participating in the alliance seek common interests while maintaining their independence (Zhang et al., 2021c).

Various factors affect the quality of logistics services. Some scholars believe that the management of internal suppliers, operation processes, and sales processes in logistics distribution centers plays a decisive role in the quality of logistics services (Clithero et al., 2021; Han, 2021). It must take customer satisfaction as the service purpose to improve the performance level of logistics distribution center personnel to optimize the quality of logistics service. Other scholars believe that logistics quality management is very important and that timeliness determines the quality of logistics services. Managers should combine quality plans with enterprise strategies, establish closer contact with suppliers, and improve quality management skills through education and training (Lee, 2021; Hermann, 2022).

The calculation method of per capita distribution in logistics distribution service is shown in Equation 1.


(1) Q=e/d

In Equation 1, *e* represents the delivery quantity, and *d* represents the number of delivery personnel. The calculation method of the delivery time ratio is shown in Equation 2.


(2) $$T=\frac{t}{\left(d\times D\times Y\right)}$$

In Equation 2, *t* represents the total delivery time, *D* represents the working days, and *Y* represents the working hours of normal shifts. The calculation method of the distribution cost rate is shown in Equation 3.


(3) $$B=\frac{\left(P+p\right)}{Z}$$

In Equation 3, *P* represents the self-vehicle distribution cost, *p* represents the external vehicle distribution cost, and *Z* represents the total logistics cost.

The calculation method of the actual wage cost difference is shown in Equation 4.


(4) L=J+G

In Equation 4, *J* represents the difference in labor efficiency, and *G* represents the difference in labor wages. The calculation method of the difference in manual efficiency is shown in Equation 5.


(5) J=(W-w)×k

In Equation 5, *W* represents the actual working hours of deliverers, *w* represents the standard working hours of deliverers, and *k* represents the standard wage rate. The calculation method of the labor wage difference is shown in Equation 6.


(6) G=(U-k)×W

In Equation 6, *U* represents the actual wage rate, and the remaining letters have the same meaning as the above equation. The calculation method of common supply days is shown in Equation 7.


(7) $$D\,S=\frac{A_{j}+\sum_{j=1}^{n}I_{j}}{\sum_{j=1}^{n}D_{j}}$$

In Equation 7, *DS* represents the common supply days of the inventory of the distribution center, *A*_*j*_ represents the number of inventory units allocated from the factory warehouse, *I*_*j*_ represents the number of inventory units of distribution center *j*, and *D*_*j*_ represents the daily demand of distribution center *j*. The calculation method of *A*_*j*_ is shown in Equation 8.


(8) $$A_{j}=\left(D\,S-I_{j}/D_{j}\right)\times D_{j}$$

In Equation 8, the letters have the same meaning as the above equation.

### Test samples and data

#### Development status of e-commerce B

To study the optimization of logistics service quality of e-commerce with multiple consumption psychology, vegetable e-commerce B is selected as the research object. E-commerce B was officially launched in June 2016, and its main business is delivering vegetables, fruits, and aquatic products to customers within its jurisdiction. According to relevant statistics, by the end of June 2020, the delivery orders of e-commerce B exceeded 10 million, making it the preferred e-commerce provider for local people (Chen and Kim, 2021; Tan et al., 2021; Pradhan, 2022; Zhan et al., 2022). The COVID-19 outbreak has seen an “explosion” in orders for the e-commerce company.

#### Research methods

**FIGURE 4:**
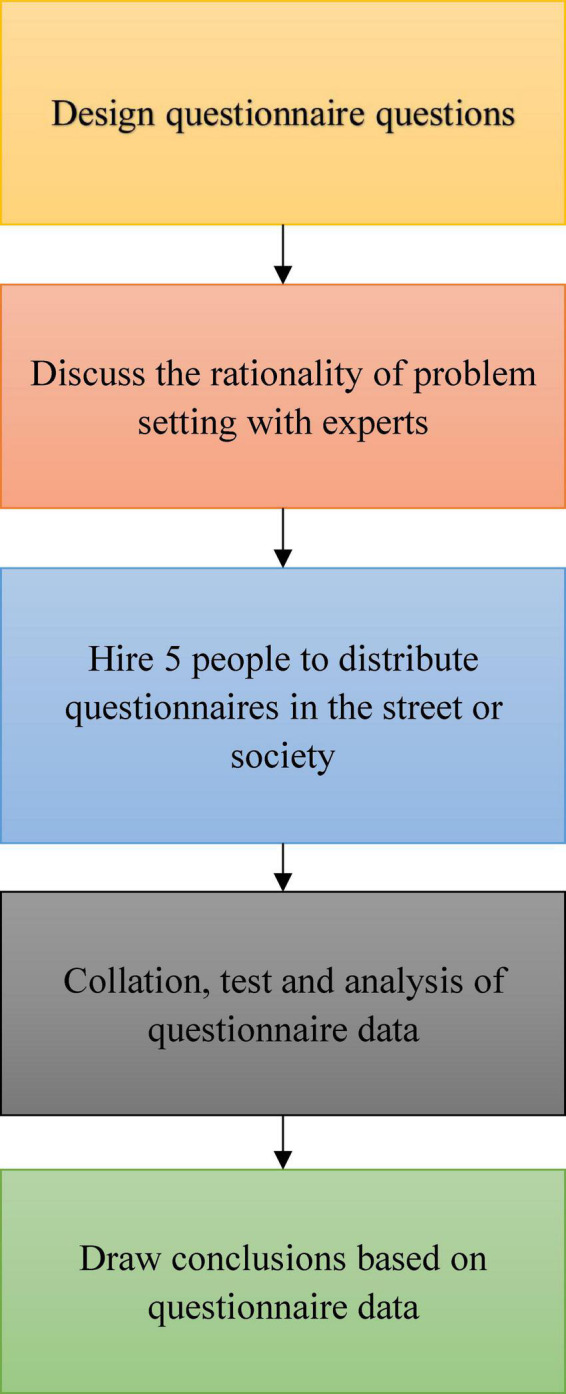
Questionnaire distribution process.

Using the method of questionnaire survey, 300 people are randomly selected to investigate. The specific process of questionnaire distribution is shown in Figure 4.

To improve the accuracy of the questionnaire results, the questionnaire is conducted in person. After the invalid questionnaires are removed, the number of valid questionnaires finally collected is 246.

#### Sample data testing

To make the results of the questionnaire more accurate, the Kaiser Meyer Olkin (KMO) validity test is performed on the data. The specific calculation method is shown in Equation 9.


(9) $$\text{KMO}=\frac{\sum \sum_{i\neq j}r_{i\,j}^{2}}{\sum \sum_{i\neq j}r_{i\,j}^{2}+\sum_{i\neq j}r_{i\,j∙1,2\,\ldots \,k}^{2}}$$

In Equation 9, *r*, *i*, *j*, and *k* represent the correlation coefficient, dependent variable, independent variable, and quantity, respectively. The specific test criteria are shown in Table 1.

**TABLE 1:** Kaiser Meyer Olkin test criteria.

Type	Range of values	Factor analysis is appropriate
KMO value	>0.9	Very much suitable
	0.8∼0.9	Quite suitable
	0.7∼0.8	Fit
	0.6∼0.7	Not very suitable
	0.5∼0.6	Barely fit
	<0.5	Unsuited

Based on Equation 9, SPSS 25.0 is used to analyze the validity of the designed questionnaire data. The KMO value is 0.869, ranging from 0.8 to 0.9 (*p* = 0) < 0.01. The results show that the questionnaire data are very suitable for factor analysis, and the questionnaire survey has good effectiveness.

In addition, the reliability of the questionnaire data is tested, and the specific calculation method is shown in Equation 10.


(10) $$\alpha =\frac{k}{k-1}\,\left(1-\frac{\sum_{i=1}^{k}\sigma_{Y\,i}^{2}}{\sigma_{X}^{2}}\right)$$

In Equation 10, *k* represents quantity, *X* represents the dependent variable, and *Y* represents the independent variable. The higher the reliability coefficient is, the higher the reliability between variables, indicating the higher internal consistency between variables. The above equation is used to test the reliability of the internal consistency of the answers to the questionnaire, and the calculated result is 0.86, indicating that the questionnaire has a high reliability.

## Results and discussion

### Logistics service quality assessment of e-commerce platform B

**FIGURE 5:**
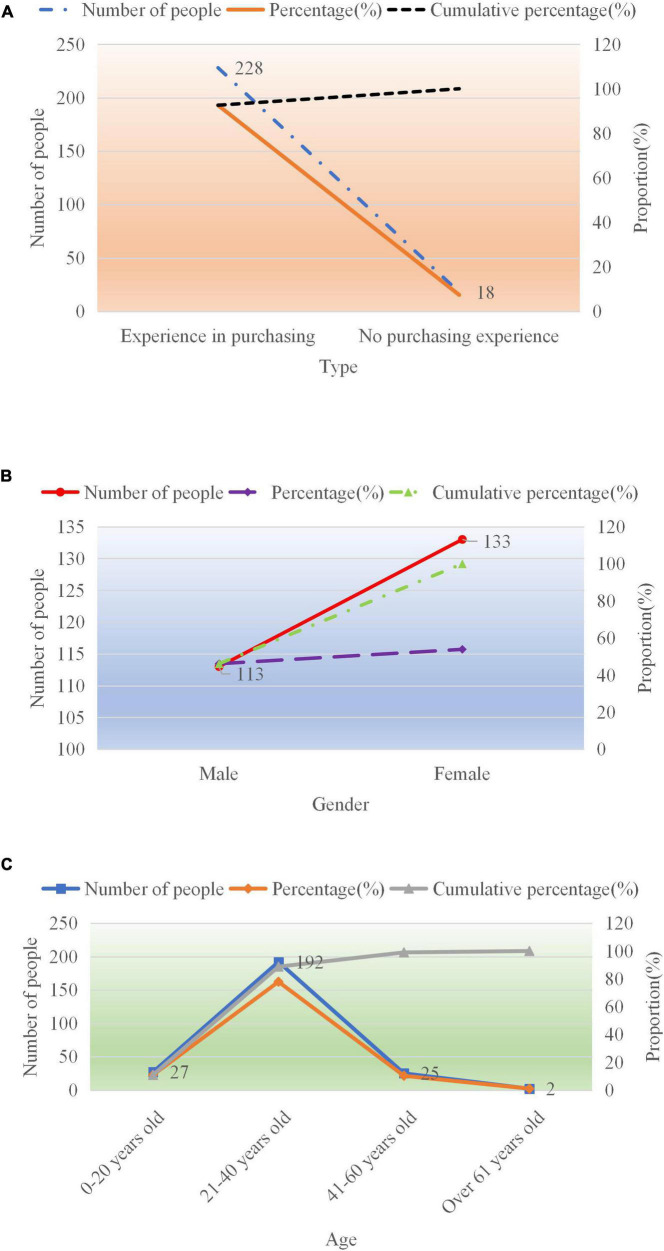
Basic information of the respondents. Panel **(A)** indicates whether the respondents have purchased the goods of the store, panel **(B)** indicates the gender of the customers, and panel **(C)** indicates the age of the customers.

The basic information of the respondents in the above questionnaire is shown in Figure 5.

From Figure 5, the customers who purchased goods from e-commerce B account for approximately 92.6% of the total number of respondents. The number of people who have not bought one is approximately 7.4%. In terms of gender, there is not much difference between men and women, with men accounting for 46.13% and women 53.87% of the total. In terms of age, customers aged 0–20 years old account for 10.86%, those aged 21–40 years old account for 77.9%, those aged 41–60 years old account for 10.24%, and those aged over 61 years old account for 1%. From these data, it is inferred that the majority of women aged 21–40 choose to shop on e-commerce platform B.

**FIGURE 6:**
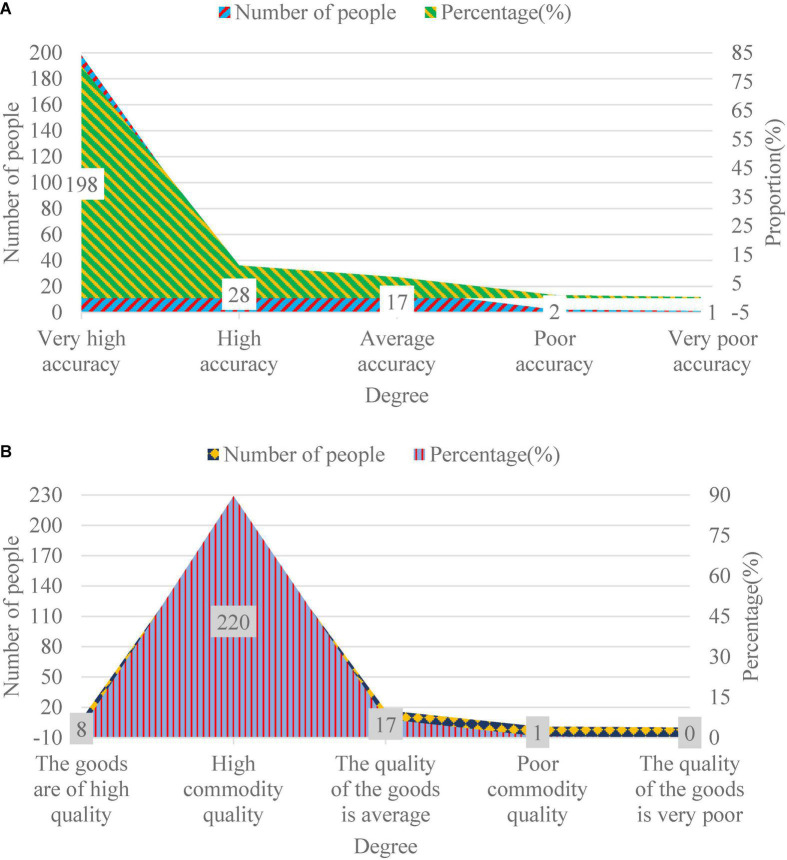
Respondents’ evaluation of the quality and accuracy of goods. Panel **(A)** shows the correctness of the quantity and type of goods purchased. Panel **(B)** shows the quality of goods.

When they are asked questions related to product quality, the specific situation is shown in Figure 6.

Figure 6 shows that when asked about the accuracy of goods, about 91.6% of customers think the accuracy of e-commerce B is high or very high, 7.18% think it is average, and 1.22% think the accuracy is poor or very poor. In terms of product quality, 92.64% of customers think that the product quality is high, and there are no problems such as rot and stink. 6.89% of customers think the quality of goods is average, while 0.47% of customers think the product quality is poor. From these data, e-commerce B pays more attention to the accuracy and quality of goods, which can bring users more satisfactory shopping experience.

**FIGURE 7:**
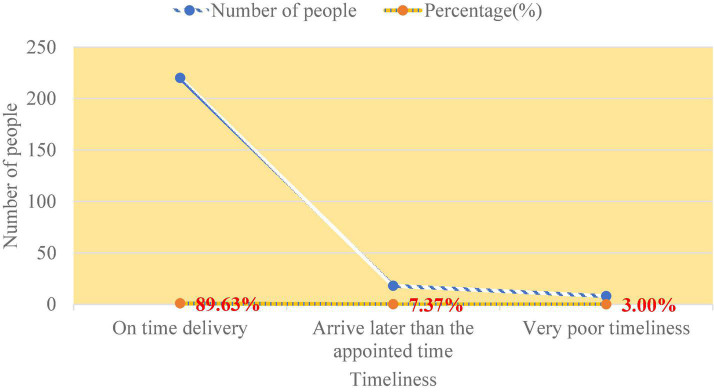
Delivery timeliness of goods on e-commerce platform B.

The results of the survey on the distribution of goods are shown in Figure 7.

From Figure 7, 89.63% of customers think delivery personnel can deliver goods on time, and 7.37% think delivery time will arrive later than the agreed time, but the impact is not significant. Three percent of customers rate its delivery timeliness as very poor. Later, we asked these 3% again, and it is found that orders with a large difference from the agreed time generally occur during the epidemic, which is caused by the surge of orders. In this way, the delivery accuracy rate of e-commerce B can basically meet consumers’ wishes.

**FIGURE 8:**
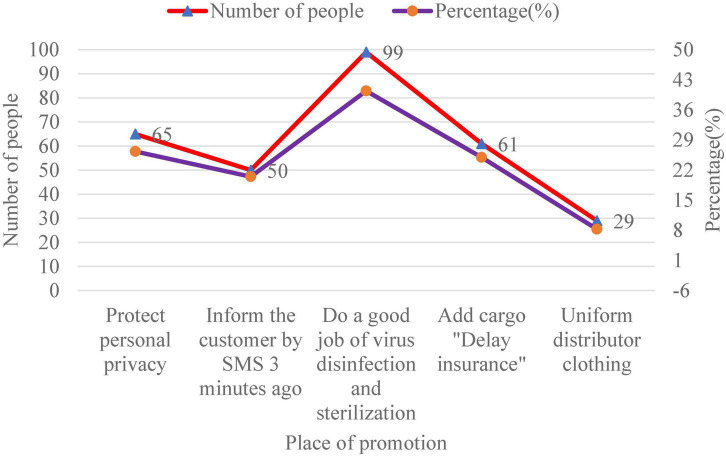
The distribution aspects of e-commerce platform B that need to be improved.

In addition, it also sorted out the respondents’ opinions on “what needs to be improved in the distribution of e-commerce platform B,” as presented in Figure 8.

**FIGURE 9:**
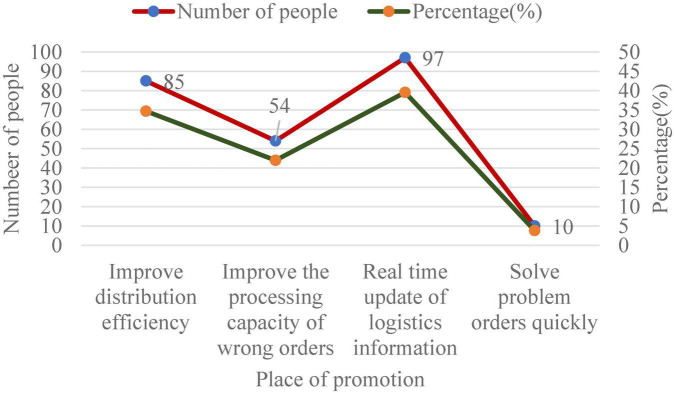
Professional aspects of e-commerce platform B that need to be improved.

Figure 8 shows that customers who want to protect their privacy account for 26.34% of the total number of customers, who want delivery personnel to inform customers by text message 3 min ago account for 20.49% of the total number of customers, and those who want delivery personnel to do a good job in virus elimination account for about 40.42% of the total number of customers. 24.97% of the total number of customers hope to add cargo “delay insurance” to the platform. Finally, 8.27% of the customers believe that the clothes of deliverers should be uniform. In addition, “what needs to be improved in the professionalism of e-commerce platform B” is shown in Figure 9.

From Figure 9, 34.68% of customers hope that e-commerce B can timely pick up and deliver goods after the information is confirmed, 21.97% of customers believe that the ability of delivery personnel to deal with wrong orders should be improved, and 39.54% of customers believe that the logistics information of purchased goods should be updated in real time. In a survey, 3.81% of customers believe that problem orders should be solved quickly. Obviously, although e-commerce platform B has done a good job in terms of product quality, accuracy and delivery timeliness, there are still many areas to be improved in the future.

### Optimization of e-commerce logistics service quality under multiple consumption psychology

Customers’ multiple consumption psychology will hold different attitudes toward e-commerce logistics services. Based on the mentioned requirements for improvement in the distribution and professionalism of customers, optimization research is conducted from the perspective of multiple consumption psychology.

#### Optimization of commodity distribution personnel management

First, in the selection of delivery personnel, company B should improve the requirements and standards. If it is implemented according to the “all take” standard, it will not only increase the company’s training costs but also may lead to complaints later. In terms of personnel selection, e-commerce B should strictly check the pass and require delivery personnel to verify their identity with face recognition before and after each order delivery to avoid impostors. Additionally, confidentiality agreements should be signed when labor contracts are signed. Finally, the prejob training and assessment of distribution personnel should be strengthened to ensure that customers have high-quality logistics service experience. In the case of force majeure on the way of distribution, such as heavy rain, car accidents, and other factors, late appeals can be carried out.

#### Improvement of basic services

Appropriate refrigeration equipment should be introduced. Because the distribution personnel will be part of the frozen goods placed in the designated place, customers sometimes have not picked up the goods in time, which will cause the goods to rot. The refrigeration unit can view the previous order attributes to weigh whether to place. It is recommended to place refrigeration equipment in busy areas because the delivery time of goods may not coincide with the customer’s leaving time, so refrigeration equipment is very important. In addition, the neatness and uniformity of clothing will give customers a good first impression. At the same time, the color and pattern of the shopping bag can also be consistent with the clothing of the distribution staff.

#### Formulate emergency plans

The formulation of emergency plans can help e-commerce B continue to operate in an orderly manner, which can help to analyze the order quantity of each period before, predict the follow-up business volume, and do a good job of allocating distribution personnel and picking personnel. These periods include holidays, around the Chinese New Year, and after the outbreak of the epidemic. Under the condition of ensuring the normal operation of business, certain rewards can be given to distribution personnel. At the same time, it is also imperative to improve the usual welfare benefits and stimulate the enthusiasm of employees.

#### Strengthen cooperation and learn from good practices

E-commerce B is gradually becoming increasingly larger. In this process, it should learn and refer to the excellent practices of other e-commerce platforms, such as Hema Xiansheng. In addition, it should also strengthen cooperation with other well-known brands and expand their business scope, not limited to the field of vegetable and fruit distribution. Opening the services of daily provisions, medicines, and errands can let more groups know about e-commerce and join e-commerce. It can hold various coupon sending and full reduction activities in holidays or special time to stimulate the enthusiasm of customers. On the way to become larger and stronger, the purpose of customers should always be kept in mind first, the efficiency of after-sales problem solving should be improved, and the common improvement of reputation and performance should be realized without losing customers.

## Discussion

With the rapid rise of logistics industry and 5G, it provides a broad platform for the development of e-commerce industry. Taking vegetable e-commerce B as the research object, questionnaire survey is performed to study its product quality, delivery speed, and aspects that consumers thought e-commerce B needs to be improved. Finally, implementable opinions are put forward from four different perspectives according to the conclusions reached. Zhang et al. (2021a) analyzed the causes of e-commerce reverse return and exchange logistics from the two aspects of defects of commodity and logistics service quality and consumers’ willingness, explored the problems of return and exchange logistics, pointed out specific solutions, and analyzed the necessity of e-commerce return and exchange logistics insurance (Zhang et al., 2021a). Dam and Dam (2021) believed that service quality depends on the gap between the actual perceived service level and the expected service level of customers. The service quality score can be calculated by subtracting the expected service score from the actual perceived service score. If the calculation result is negative, the customer is not satisfied with the service quality, otherwise, the customer is satisfied. The SERVQUAL model has been widely used by service scholars and has become mature, but scholars’ research on SERVQUAL is continuing (Dam and Dam, 2021). The two scholars studied the development of e-commerce from two aspects of return and exchange logistics and service quality, respectively, but there are few aspects involved and there are limitations. This study is more comprehensive and complete. However, the disadvantage is that the logistics of return and exchange has not been carefully studied, and the research method is relatively single. Therefore, follow-up research should be strengthened.

## Conclusion

The emergence of Internet of Things technology provides a broad space for the development of e-commerce logistics. From the perspective of multiple consumption psychology, optimization research is implemented on the quality of e-commerce logistics service, and the following conclusions are reached. Firstly, vegetable e-commerce supplier B is selected as the research object, and the quality, accuracy, and improvement of its delivered goods are studied by questionnaire survey. Second, 91.6% of customers think that the accuracy of e-commerce B is high or very high, 92.64% of customers think that the quality of goods is high and there are no problems such as rot and stink, and 89.63% of customers think that deliverers can deliver goods on time. This shows that the overall service level of the platform is relatively high. Third, according to the survey of the surveyors on “what needs to be improved in terms of distribution and professionalism of e-commerce platform B,” there are still many areas to be improved, such as privacy of customer information, security of goods, and timely update of logistics information. Fourth, based on the above problems, the author puts forward optimized opinions and suggestions from four different perspectives of multiple consumption psychology.

Due to limited energy, only the logistics service quality optimization of vegetable and fruit e-commerce is studied, while other types of e-commerce are not analyzed. The research methods are mainly from the perspective of multiple consumption psychology, and the aspects involved are limited. In addition, the data in this work only come from offline questionnaire survey, which has certain limitations. In the follow-up research, both online and offline can be considered to conduct all-round and multi-field research on various type of e-commerce, so as to promote the rapid development of e-commerce industry.

## Data availability statement

The raw data supporting the conclusions of this article will be made available by the authors, without undue reservation.

## Author contributions

MM and LS conceptualized the study and wrote the draft of the manuscript. MM and XS validated the data used and edited the manuscript. All authors contributed to manuscript writing, read, and approved the submitted version.
